# Assessment of Biomechanical Predictors of Occurrence of Low-Amplitude N1 Potentials Evoked by Naturally Occurring Postural Instabilities

**DOI:** 10.1109/TNSRE.2022.3154707

**Published:** 2022-03-08

**Authors:** Rahul Goel, Sho Nakagome, William H. Paloski, Jose L. Contreras-Vidal, Pranav J. Parikh

**Affiliations:** Department of Health and Human Performance, University of Houston, Houston, TX 77204 USA; Department of Electrical and Computer Engineering, University of Houston, Houston, TX 77004 USA.; Human Research Program, NASA Johnson Space Center, Houston, TX 77058 USA.; Department of Electrical and Computer Engineering, University of Houston, Houston, TX 77004 USA.; Department of Health and Human Performance, University of Houston, Houston, TX 77204 USA

**Keywords:** Center of pressure velocity, fronto-central negativity, N1 potential, naturally occurring postural instability, time to boundary

## Abstract

Naturally occurring postural instabilities that occur while standing and walking elicit specific cortical responses in the fronto-central regions (N1 potentials) followed by corrective balance responses to prevent falling. However, no framework could simultaneously track different biomechanical parameters preceding N1s, predict N1s, and assess their predictive power. Here, we propose a frame-work and show its utility by examining cortical activity (through electroencephalography [EEG]), ground reaction forces, and head acceleration in the anterior-posterior (AP) direction. Ten healthy young adults carried out a balance task of standing on a support surface with or without sway referencing in the AP direction, amplifying, or dampening natural body sway. Using independent components from the fronto-central cortical region obtained from subject-specific head models, we first robustly validated a prior approach on identifying low-amplitude N1 potentials before early signs of balance corrections. Then, a machine learning algorithm was used to evaluate different biomechanical parameters obtained before N1 potentials, to predict the occurrence of N1s. When different biomechanical parameters were directly compared, the time to boundary (TTB) was found to be the best predictor of the occurrence of upcoming low-amplitude N1 potentials during a balance task. Based on these findings, we confirm that the spatio-temporal characteristics of the center of pressure (COP) might serve as an essential parameter that can facilitate the early detection of postural instability in a balance task. Extending our framework to identify such biomarkers in dynamic situations like walking might improve the implementation of corrective balance responses through brain-machine-interfaces to reduce falls in the elderly.

## I. Introduction

Posture control during upright stance is one of the most fundamental motor tasks in everyday life. It requires minimum energy with almost no mental effort. Deterioration and impairment of this essential human skill, for instance, with aging, may cause a physically dangerous and sometimes fatal fall. There is a moment of postural instability preceding every fall. The characterization of that moment is critical and could help us better understand how the central nervous system (CNS) predicts future postural instability.

Under normal conditions of an upright stance, the CNS balances the body by maintaining the vertical projection of the center of mass (COM) at a quasi-steady equilibrium point near the center of the base-of-support, called the center of pressure (COP) [1]. To do so, the CNS continuously monitors the afferent sensory information from various receptors (e.g., cutaneous receptors, muscle stretch sensors, joint receptors, vestibular end-organs, and visual sensors) and determines the state of standing balance [2]. In case of a deviation from the equilibrium, it adjusts the motor commands to bring the COM back to the equilibrium point. An alternative view [3] holds that the underlying objective for controlling posture is not to minimize departure from the stability point within the equilibrium region but to reduce the deviation of sway toward a stability limit. In other words, the CNS may utilize the temporal and spatial dynamics of the COP concerning its stability boundaries to characterize postural instability [4], [5].

The frontal and parietal cortical regions are known to be involved in the control of posture under challenging environmental conditions that may lead to a fall [6]–[30]. In our previous work [15], we used MRI-constrained electroencephalography (EEG), and MRI-guided inhibitory theta-burst transcranial magnetic stimulation over the supplementary motor area (SMA), while subjects performed a balance task with varying stability of the support surface. We found changes in EEG spectral power within anterior cingulate gyrus, cingulate gyrus, and posterior parietal cortex areas along with alterations in the control of posture during the challenging standing task in the theta-burst group compared to the control group, which received sham stimulation [15]. These findings suggested that the cortical network involving fronto-parietal regions might be involved in monitoring ongoing postural responses and performing suitable adjustments to the motor commands if a deviation from stability is detected such that we are not catching up with instability after it has occurred [31].

Several studies [11], [13], [32] have attempted to identify an early cortical sign of postural instability. A negative potential (N1) generated over the fronto-central region, around 100–200 ms following naturally occurring instability while standing still [14] or following external perturbation of the support surface [6], [7], [9], [10], [17], [19], [21], [22] has been suggested to indicate the involvement of higher-order processing in the form of detecting an error due to the difference between anticipated and actual postural states and signaling functional postural responses, if necessary [9], [10], [13], [27], [32]. These findings suggest that the occurrence of N1 would always indicate a presence of postural instability, regardless of its cause. This advanced knowledge of an imminent fall can be helpful in populations at high risk of falls to produce corrective neuromuscular responses early on through intrinsic mechanisms and/or to produce corrective response through a body-worn exoskeleton [21]. However, for such brain-machine applications, one important consideration is how early we can detect the cortical reaction [19], [21] so that we can intervene.

Detection of a cortical reaction following a naturally occurring postural instability is difficult. This is because the N1 potential is small in size and, therefore, is technically challenging to detect beyond the ongoing EEG activity [14]. To our knowledge, there is only one study that attempted to reliably detect such an N1 response during a continuous standing task where there is no fixed perturbation onset. Varghese *et al.* [14] located the low amplitude N1 response using the changes in biomechanical parameters assessed during the corrective balance response, i.e., using parameters following the N1. However, for practical applications, it is desirable to predict N1 responses during a naturally occurring balance perturbation using biomechanical parameters that precede and not succeed, such a cortical response.

Various biomechanical parameters may serve as a control variable predicting the cortical signature of postural instability. For instance, Slobounov *et al.* found that the time to contact the postural stability boundary called time to boundary (TTB) and not standard COP measures were related to EEG response during a postural stability task using separate statistical analyses [13], [33]. Other studies have suggested parameters such as surface stability [11], COP excursions [14], ground reaction forces [34], COM acceleration [35], and COM velocity [36], [37] as possible control variables that the CNS tracks for the control of posture. However, none of these studies investigated what biomechanical parameter among several parameters during a given trial or epoch best predicts the occurrence of a cortical response. This requires comparing the ability of different biomechanical parameters to predict a cortical reaction during a given trial/epoch. Such a direct comparison will elucidate the most important biomechanical parameter that triggers a cortical response following postural instability.

Therefore, the primary aim of this study was to develop a framework that can be used to assay biomechanical parameters in single trials and predict the occurrence of low-amplitude N1 in healthy young adults from the tracked parameters. We achieved this by developing machine learning algorithms to predict the occurrence of low-amplitude N1 potential from single epochs of biomechanical parameters. Next, we examined the impact of different biomechanical parameters on the outcome of machine learning models.

## II. Methods

### A. Participants

Ten (4 females) healthy right-footed young adults (mean SD, age: 25.3 ± 3.3 years; height: 167.7 ± 12.8 cm; and weight: 67.5 ± 15.5 kg) provided informed written consent to participate in this study. Subjects were included in the study only if they were in the age range of 18–35 years and had a body mass index of less than 30 kg/m^2^. They had no history of balance, neurological, musculoskeletal, movement, cardiovascular, or vestibular disorders and were not using any medication known to impact neuromuscular functions. The study was approved by the University of Houston Committee for the Protection of Human Subjects.

### B. Instrumentation

#### 1) Computerized Dynamic Posturography (CDP):

We utilized a standard CDP force platform (Neurocom Balance Manager, Natus Medical Incorporated, Pleasanton, CA) to assess the stability of the posture control system. The Neurocom posturography system has been used in research [15], [17], [38], [39] and clinical [40] settings to assess the motor and sensory performance of the posture control system. A dynamic dual force plate system (45.72 cm × 45.72 cm) is inbuilt in the CDP platform and can be controlled to sway or translate in the AP direction only. Four individual force transducers within the force plate collected ground reaction forces from under the subjects’ feet. A shear force sensor collected SH in the AP direction. All the ground reaction force data were collected at 100 Hz. Pre-installed software on a Windows-based desktop connected to the Neurocom Balance Manager (Research module, Neurocom software v8.0, Natus Medical Incorporated, Pleasanton, CA) processed the data. An analog output signal of 5 V was produced by the Neurocom system at the start of each trial for synchronization with other systems.

#### 2) Electroencephalography (EEG):

Whole-scalp EEG data were collected by active 64 channel EEG electrodes (Brain Products GmbH, Germany) at a sampling frequency of 1000 Hz. Modified International 10–20 system was used for electrode placement [41]. The left earlobe was used as the ground electrode, whereas the right earlobe was used as the reference electrode. A camera-based 3D scanning system (BrainVision Captrak, Brain Products GmbH, Germany) was used for digitizing the EEG electrodes’ spatial position.

#### 3) Accelerometer:

A wireless inertial motion unit (IMU) called OPAL sensor (APDM Inc., Portland, OR) was mounted on the subject’s head, recording three-axis accelerations and angular velocities at 80 Hz. However, only head acceleration (HA) in the AP direction was used in our analyses.

### C. Experimental Procedure

All subjects participated in two sessions, around a week apart. The first session was used to assess baseline postural performance using standard sensory organization tests (SOTs) of the Neurocom posturography system, record body weight, height, foot size, head circumference, and obtain structural brain MRI. High-resolution T1-weighted structural images were obtained using a 3T Siemens Trio whole-body MR scanner (Erlangen, Germany) at the Center for Advanced MRI (Baylor College of Medicine, Houston, TX).

**Fig. 1.**
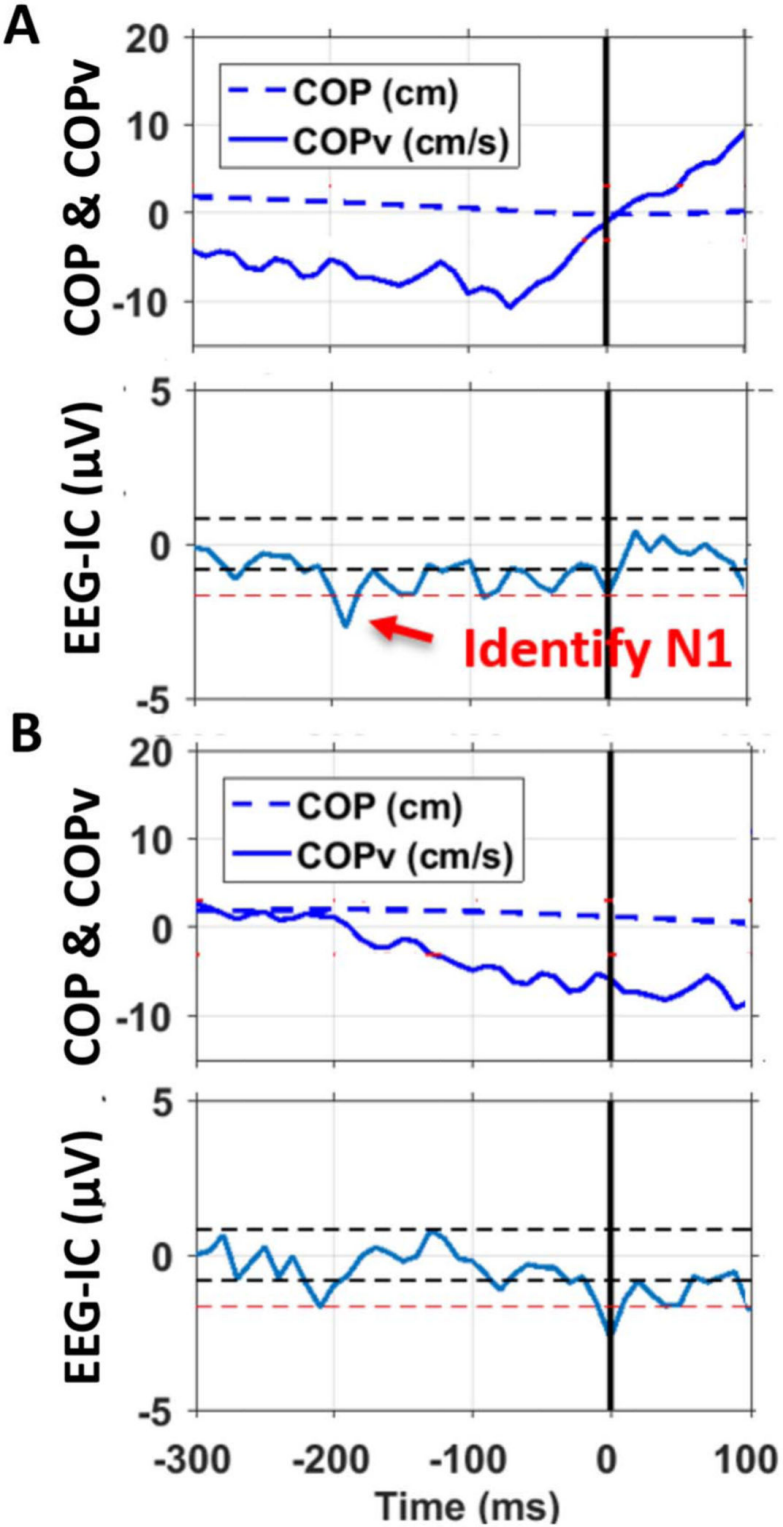
(A) Top Panel: Detecting zero-crossing (time = 0) in COPv following a large amplitude peak in COPv (~ time = − 70 ms), Bottom Panel: Instability-evoked N1 time-locked (time = 0) to change in sign of COPv. Black horizontal dash lines are 1SD around the mean, whereas the red dash line is 2SD from the mean; (B) Top Panel: COP and COPv time series time-locked (i.e., re-centered, time 0) at the selected N1, and an epoch of −300 ms to 100 ms around N1 was saved. However, only data from −300 to −50 ms was used in further analyses. Bottom Panel: The EEG IC time series time-locked (i.e., re-centered, time = 0) at the selected N1. It is to be noted that time = 0 in top two and bottom two panels are not the same time points in reality.

The second session was the primary experimental session, in which subjects were first instrumented with EEG electrodes and an IMU in the middle of the frontal bone above the eyebrows. One continuous balance task lasting 180 s and consisting of nine consecutive 20 s long balance trials were performed, without any rest in between, with varying surface stability conditions with eyes closed. EEG, ground reaction forces, and HA were measured and analyzed during the balance task. The support surface was sway-referenced by tilting in the AP direction in various proportions to the estimated instantaneous COM sway angle in the sagittal plane with negligible time delay. It means that the support surface would sway if the subject swayed, amplifying or dampening naturally occurring body sway. Three trials had a gain of 0 (i.e., quiet stance), whereas the gain varied for the other six trials. All subjects underwent the task in the following order of gains: 0, 0.4, 2.0, 0, 0.6, −0.4, 0, 1.0, −1.0 (see Fig. 1 in our previous publication [15], provided as Supplementary Fig. S1). The first trial (a quiet stance with eyes closed) started ~10 s after subjects were told to close their eyes to avoid any transient effects and collect baseline (pre-task) data for EEG mean correction. Subjects were unaware when the gain of the support surface changed. Different balance conditions within the single continuous standing balance task allowed us to naturally manipulate the difficulty of the posture control system while concurrently monitoring changes in ground reaction forces, HA, and EEG. Subjects were connected to a loose safety harness to prevent falls or injury during the posture task. Subjects were not allowed to practice the balance task with sway referencing.

### D. Data Analyses

Ground reaction force data collected from the vertical force sensors were first combined to create COP time series in the AP direction [42]. COPv was estimated from the COP using a three-point central difference algorithm [15], [17], [43] in MATLAB 2017a (Mathworks, Natick, MA), which does not introduce any time delay. Anterior and posterior anatomical foot limits were used to create a rectangular stability zone for each subject. The instantaneous differences between the stability boundaries and the AP COP position in COP movement (anterior or posterior) direction were estimated as the distance to boundary (DTB). Time to the boundary (TTB) was calculated by dividing DTB by the COPv magnitude [15], [16], [18].

As done in a previous study, the COPv in the AP direction was used to identify early signs of discrete balance corrections to postural instabilities [14]. Specifically, large amplitude peaks in COPv greater than 3SD of COPv during the first 20 s quiet stance trial (when gain was 0) were selected (see the lowest COPv point ~ time = −70 ms in the top panel of Fig. 1A) and used to find the subsequent zero-crossing in the COPv time series (see ~ time = 0 in the top panel of Fig. 1A). The zero-crossing in COPv also coincides with the time point of the peak in COP position and represents a biomechanical marker of an early sign of corrective balance reaction to postural instability (see Fig. 1 in [14]). The amplitude of the peak COPv threshold was adjusted for each subject but was typically around 6 cm/s. Raw SH and HA in the AP direction were down-sampled to 10 Hz for further analyses.

Steps used for EEG pre-processing are explained in our prior publications [15], [17]. In brief, the raw EEG signal collected at 1000 Hz was first down-sampled to 100 Hz and then high-pass filtered at 0.1 Hz using a 4^th^ order Butterworth filter. A standardized EEG processing pipeline (PREP) was used [44] to apply common referencing methods and remove artifactual EEG channels. Next, Artifact Subspace Reconstruction was applied [45] to identify and remove the artifactual principal components and then reconstruct the signals in the EEG data. Next, we ran the independent component analysis (ICA) using adaptive mixture ICA (AMICA) [46]. After that, a dipole fitting method (DIPFIT in EEGLAB) was used to calculate the equivalent current dipole sources that explain at least 85% of the topographic variance obtained from ICA with subject-specific head models created from MRI scans from individual subjects and digitized EEG channel locations. The EEG time series were reconstructed using the independent components (ICs) remaining after removing dipoles located outside the individual head models or those that had artifactual components (e.g., muscle related power spectral density characteristics, motion artifact related high-frequency noise, etc.).

**Fig. 2.**
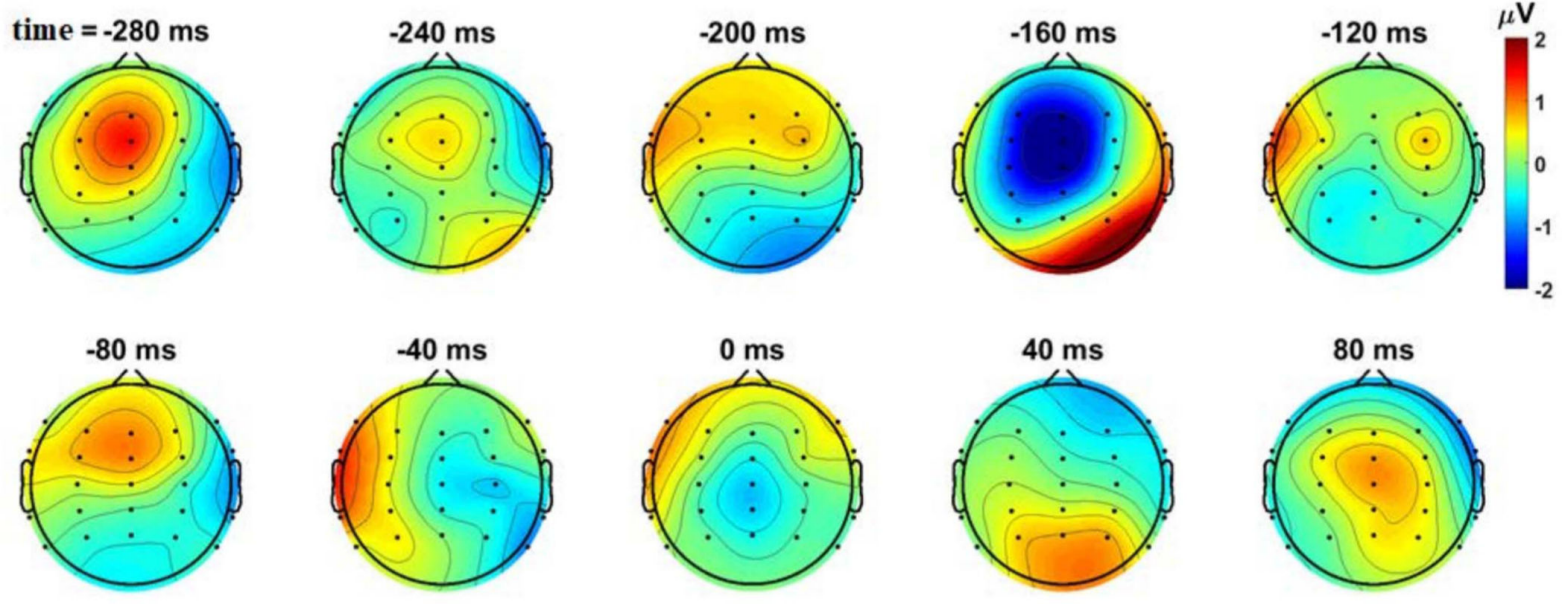
Scalp topographies of naturally occurring instability-evoked cortical response at selected time points during the balance task time-locked (i.e., time = 0) to the change in sign of COPv for a representative subject (averaged across 24 epochs). A significant negative depression is visible at −160 ms around the fronto-central region, for this subject.

Then a representative IC over the fronto-central region was selected by looking at both the two-dimensional topoplots of all the ICs remaining after EEG data cleaning and the corresponding dipole projections in three dimensions, for each subject. This technique of choosing an IC from the fronto-central area has been validated in our previous publication [17] (see Fig. 2 in our previous publication [17], provided as Supplementary Fig. S2). But it should be noted that in this study, new ICs were identified per subject by analyzing the EEG data from the balance task with varying stability of the support surface, and these were not the same ICs identified from the perturbation tasks’ analyses as in our previous study [17]. A baseline correction was employed by subtracting the pre-task mean (of −5 to 0 s) EEG activity from the entire time series of the selected IC to control inter-subject differences. Once an IC is selected, naturally occurring postural instability-evoked N1 was identified in the temporal data of that IC as the largest negative peak, which was lower by at least 2SD (see bottom-most red horizontal dash line in the lower panel of Fig. 1A) of the baseline (pre-task mean), and was within −250 ms to −100 ms time window before the change in the sign of COPv (time = 0 in the lower panel of Fig. 1A). The temporal data was then time-locked (i.e., re-centered) at the selected N1 (Fig. 1B), and an epoch of window size −300 ms to +100 ms around N1 was saved for further analyses. Four biomechanical parameters of interest: COPv, SH, HA, and TTB, in that epoch were also saved. A total of 338 such epochs were obtained from all ten subjects from the balance task.

We visualized the topographical distribution of naturally occurring postural instability evoked N1 for each subject. Figure 2 shows the mean of baseline-corrected EEG activity (cleaned EEG at sensor level) for 24 epochs when plotted for every 40 ms from −280 ms to +80 ms, time-locked to the change in the sign of COPv (same time axis as in Fig. 1A), for a representative subject. It shows significant negative depression at −160 ms around the fronto-central region for this subject. These topoplots are similar to those published previously (see Fig. 2 in [14]), but obtained using more robust EEG pre-processing, including subject-specific head models using MRI and digitized EEG channel locations.

To make sure that we were not randomly selecting N1 as any prominent negative peak in the IC which was lower than 2SD of baseline, all the other negative peaks in the temporal data of the selected IC, which had a magnitude lower than 2SD of baseline, were also chosen, and −300 to +100 ms long epochs around these control peaks were also saved. We called the first data set the *Instability* data set (class label: 1), and the latter the *Control* data set (class label: 0). The number of epochs we could find for the *Control* data set was nearly double that for the *Instability* data set (726 vs. 338, respectively), across all subjects. The proportion of the epochs belonging to class label 1, with respect to all epochs of both classes together, had a mean (standard error [SE]) of 32.0 (3.5) %, across all subjects.

### E. Machine Learning and Statistical Analyses

Once we validated the approach to identify the naturally occurring postural instability evoked N1 potential, we were interested in identifying a single biomechanical parameter (COPv, SH, HA, TTB) or the combination of all parameters, collected before the low-amplitude N1 potentials, that could be the best predictor of the occurrence of N1s (i.e., the *Instability* data set) for our task. Thus, first, we took the median of the absolute values of the four biomechanical parameters (COPv, SH, HA, TTB) in the window −300 to −50 ms before the N1 (Fig. 1B) for each epoch in both data sets, across all subjects. We performed a similar analysis using the mean instead of the median of the absolute values of the four biomechanical parameters and obtained similar results. We chose to report the findings using the median as it is not influenced by outliers. The “end” of the window before N1 was extended only up to −50 ms and not to 0 ms as this is the order of latencies from the vestibular end-organs [47]. In our preliminary analyses, we tested with a few time points as the “start” of the window before N1, in steps of 25 ms, primarily between −350 to −250 ms relative to N1, and window lengths in 200–300 ms range, but did not find any significant difference and thus decided to choose −300 ms as the “start” of a 250 ms window before N1 (Fig. 1B), as is neuro-physiologically reasonable and technically feasible for BMI applications [19], [21]. As a preliminary step, we ran some descriptive statistics (paired ttests). We estimated the effect size (Cohen’s d) of the grand mean of differences in the four parameters across the two data sets in the −300 to −50 ms window before the N1.

We were interested in identifying which parameter will contribute most to the correct classification of the *Instability* data set. Typically, tree-based classifiers are good to understand feature importance. Therefore, several popular tree-based algorithms like Random Forest, Decision Tree, Extreme Gradient Boosting (XGBoost), Adaptive Boosting (AdaBoost), and Extra Trees were initially considered with default hyper-parameters on 20% of data, drawn five times randomly from the entire data. This is a standard technique in machine learning as every algorithm suits some problem types better than others. Consistently, XGBoost [48] provided slightly better accuracy and was fast. Gradient boosting provided the best performance in another study to predict vestibular dysfunction from postural instability [49]. Thus, we decided to apply XGBoost on the entire data with hyperparameter optimization, using the leave-one-subject-out cross-validation approach.

In this approach, results are obtained from models trained exclusively on data, not from the subject being tested on. For a left-out subject, we first created a test subset consisting of an equal number of samples of both classes by randomly undersampling the majority class. Creating a balanced test set was not essential, but it makes results convenient to compare with 50% performance metric expected by chance. For training, we combined the other nine subjects’ data and then randomly undersampled the majority class in that data, such that both classes had an equal number of training samples. Then we followed a 5-fold cross-validation (using *GridSearchCV*) approach to find the best set of hyperparameters for XGBoost (depth: 2 to 10; the number of estimators: 60 to 220; learning rate: 0.001, 0.01, 0.1) on the training data, which were then applied to the test subset created from the left-out subject’s data. This process (create test set with balanced classes from a left-out subject, undersample the majority class in the remaining data of the other nine subjects, optimize hyperparameters using 5-fold cross-validation on this balanced training data from nine subjects, apply the best hyperparameter on the test subset of the left-out subject), was repeated ten times for each of the ten subjects to obtain the results presented here so that all the data of each subject have had a chance to be in the test subset once. Accuracy, sensitivity, and area under the receiver operator curve (AUC) were the performance metrics calculated for each of the five machine learning models, i.e., each of the four parameters individually and all four parameters combined.

Further, when combining parameters for classification, it is essential to ensure that the parameters are not strongly correlated. After checking that the data were normally distributed (using Shapiro-Wilk test: *p* > 0.05), we calculated the Pearson product-moment correlation coefficients between all six possible pairs. The coefficients with COPv were: 0.34 for SH, 0.44 for HA, and −0.28 for TTB; with SH were: 0.19 for HA and −0.18 for TTB; and that between HA and TTB was −0.15. Although all correlations were statistically significant (*p* < 0.001), they were not strong (*r* < 0.7). Therefore, we were able to use them all together as parameters in the machine learning model. Further, we used SHapley Additive exPlanations (SHAP, [50]), a game-theoretic approach, to look at feature importance for the machine learning models using all four parameters.

Separate independent t-tests were used to compare the performance metrics between individual parameters and the combination of parameters. Statistical significance level was set at *p* < 0.005 (as there were ten comparisons) for the machine learning metrics and *p* < 0.05 for other descriptive comparisons. SPSS version 21 (SPSS Inc., Chicago, IL) was used for all statistical analyses.

## III. Results

**Fig. 3.**
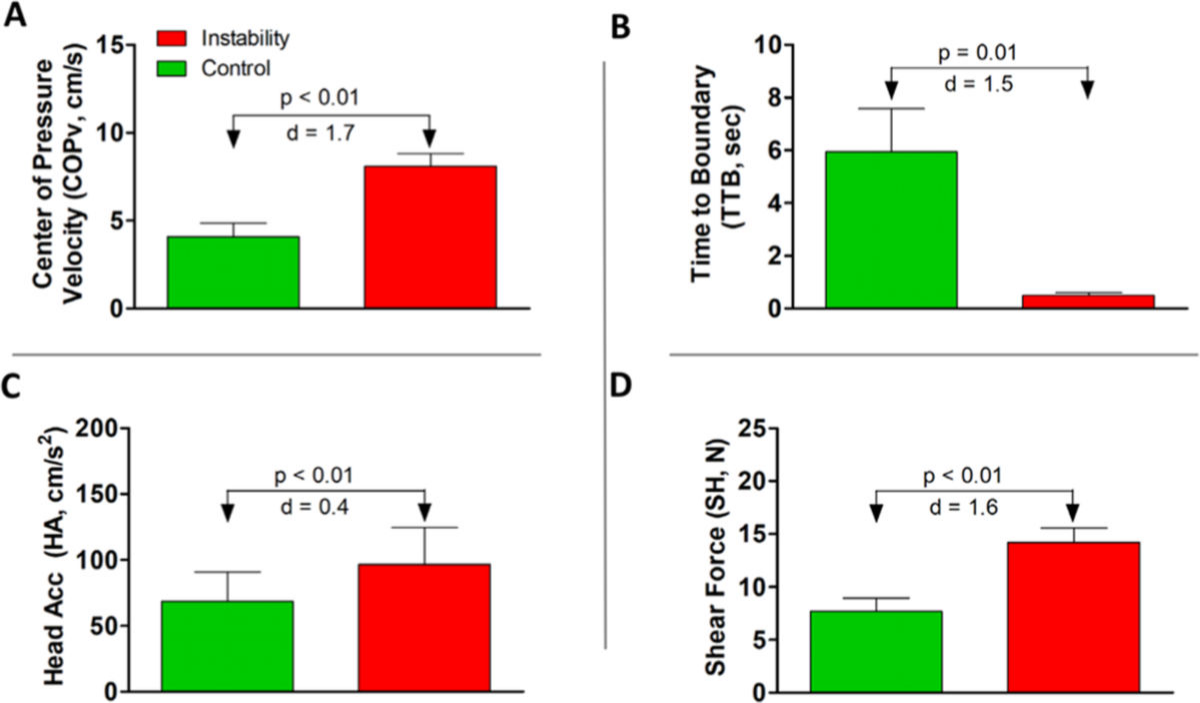
Grand means across all epochs of all subjects (median of absolute value within each epoch, where epoch is defined as the data points between −300 to −50 ms before N1) for the two data sets: (A) COPv, (B) TTB, (C) HA, (D) SH. d represent Cohen’s effect size. Error bars represent SE.

There was no report of falls. The average latency of the identified N1 potential was 164.3 ± 10.4 ms (mean ± SE), before the early sign of balance correction (change in the sign of COPv), across all ten subjects. Figure 3 shows the grand means (mean ± SE) for descriptive statistics on the four parameters calculated across all epochs, for all the subjects, along with the effect sizes. All the four parameters were significantly different (p < 0.01) across the two data sets, with very large effect sizes (Cohen’s d > 1) for three parameters (COPv, SH, and TTB).

Table I shows the three machine learning performance metrics (chance was 50%), for the binary classification, with each of the four individual and all four parameters combined. Tables II–IV show the uncorrected p-values for various comparisons for the three metrics. Using all four was typically significantly better than using each of the individual parameters, as per all three metrics. When comparing individual parameters, there was no difference between TTB and COPv. Still, both were typically significantly better than HA or SH, for all three metrics. Between SH and HA, SH was significantly better than HA in all three metrics.

### Parameter Importance:

**Fig. 4.**
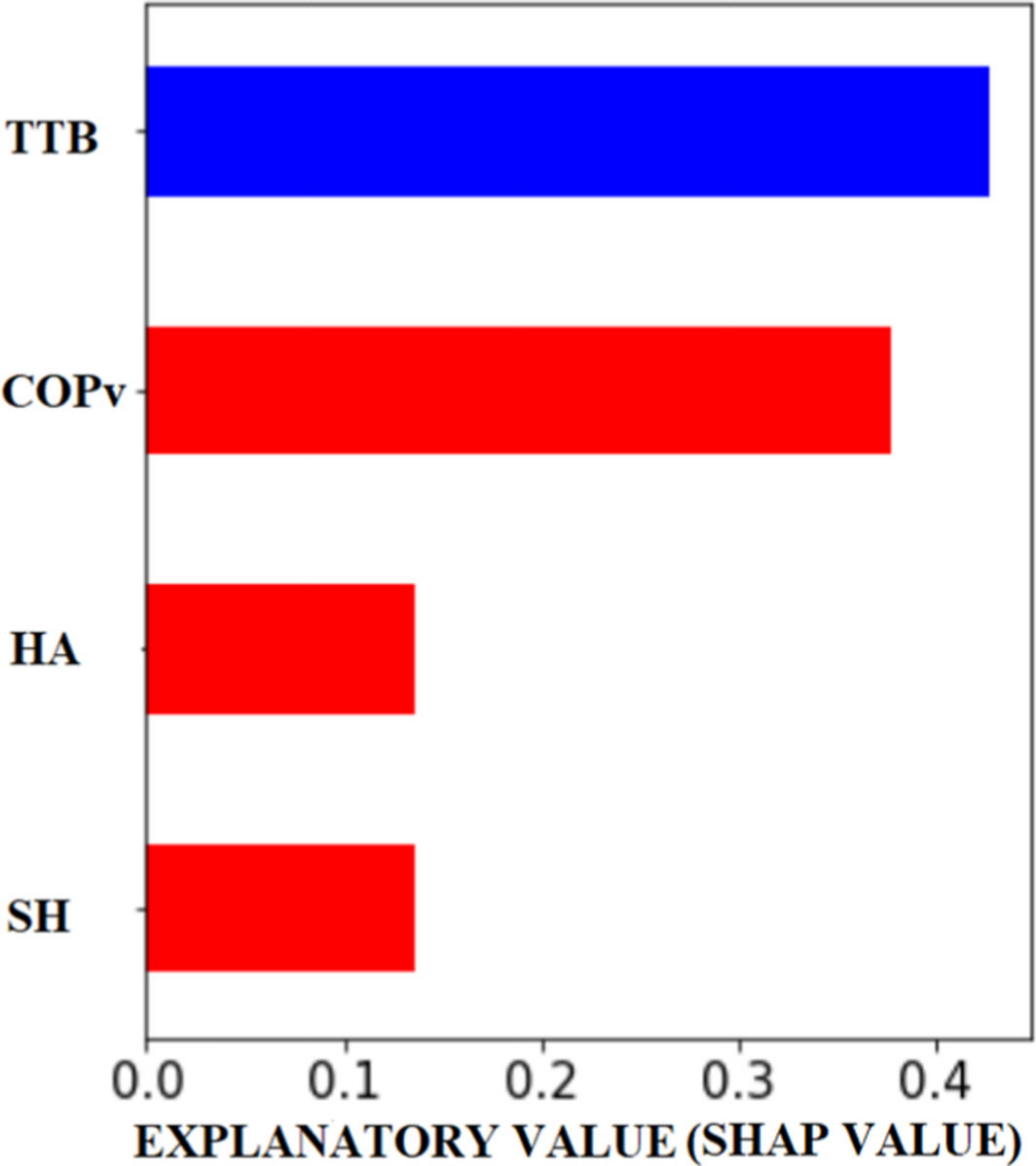
Ranking in order of importance for the four parameters when used together based on mean of |SHAP| value (average impact on model output, Red means higher values of the parameter had positive impact, blue means lower values of the parameter had positive impact).

Figure 4 shows explanatory SHAP values based on the parameter’s importance when all four were used together. TTB, on average, contributed more towards model output than COPv, whereas each of HA and SH’s relative contribution was nearly one-third of each of TTB and COPv. Values of each parameter may have a +ve or −ve impact depending on their SHAP value. As expected, higher values of COPv, SH, and HA had a positive impact on the model output (i.e., they predicted a higher probability that the model will predict the occurrence of upcoming low-amplitude N1 potentials). In contrast, lower values of TTB predicted a higher probability of occurrence of upcoming low-amplitude N1 potentials.

## IV. Discussion

In this standing balance study, we implemented tree-based machine learning models that utilized data from a single epoch to understand which of the four selected biomechanical parameters can predict the occurrence of a low-amplitude N1 potential that is typical following a naturally occurring instability. Importantly, using SHAP, a game-theoretic approach, we directly compared the importance of TTB, COPv, HA, and SH parameters. We found that TTB was the best predictor of the occurrence of low-amplitude N1 potentials when all four parameters were simultaneously considered. This result suggests that, for naturally occurring instabilities, the spatial and temporal proximity of the center of pressure to the stability boundary is the most crucial variable triggering the cortical sign of postural instability. In contrast to previous studies that obtained similar findings [13], [18], [33], [39], [51], [52], our findings resulted from a direct comparison of the prediction ability of four biomechanical parameters during a single epoch. While we showed the utility of this approach in the context of a standing balance task, it can be extended to other dynamic situations like walking.

Our previous study [17], on posture control during unpredictable external perturbation tasks, on the same subjects as in this study (perturbation tasks were carried out right after the continuous balance task in the same session), showed that the evoked N1 potential appears around (range) 150 – 215 ms before an early sign of corrective balance response (quantified as a change in the sign of COPv) in the fronto-central region. Whereas in the balance task used here, we found them with a mean around 165 ms before the same early sign of correction of balance responses. Also, to the best of our knowledge, only one study has shown cortical involvement in the form of an evoked low-amplitude N1 potential in the Cz electrode before the early sign of balance correction to naturally occurring postural instability during a quiet balance task with closed eyes [14]. Building upon these prior works, and to test our proposed framework, we first identified early signs of balance correction to large deviation in COPv in our balance task. We then identified low-amplitude N1 potential in the −250 to −100 ms window before the early signs of balance correction. We found pronounced cortical negativity (N1) in the fronto-central region before the early signs of balance correction (change in the sign of COPv) in the balance task with or without somatosensory distortion. These N1 peaks were temporally time-locked to the early signs of balance corrections. The latency of early signs of compensatory balance responses following N1 peaks was in the range of 150 – 200 ms, which agrees with the temporal course of involuntary or voluntary responses to the ongoing somatosensory inputs [17], [53], [54]. It should be noted that the magnitude of N1 evoked during our balance task where the exact time of perturbation is relatively small in comparison (around −2 *μ*V versus −10 *μ*V) to that elicited during the external perturbation task (see Fig. 4 in our previous publication [17], provided as Supplementary Fig. S3). Therefore, it is technically challenging to detect N1 potential beyond the ongoing EEG activity [14]. It is important to note that this distinction would be even more significant in older adults, who have smaller N1s than young adults for the same disturbance [55].

The fronto-central ICs that were selected to identify N1 activity were slightly on the left hemisphere (Fig. 2). This is not surprising as all our subjects were right-footed. Prior studies have postulated functional asymmetry between motor areas, with the dominant hemisphere playing a critical role in selecting appropriate postural strategies [56] and sensing a loss of balance during walking [32]. In another study, Taubert *et al.* found that improved postural performance after six weeks of balance training in right-footed individuals was associated with an increase in gray matter volume in the left SMA [57]. Overall, our results confirm the presence of N1 before the early signs of balance corrections, even during balance tasks with or without somatosensory distortion, extending the results of Varghese *et al.* [14], but using a more robust EEG preprocessing pipeline involving subject-specific head models using MRI and digitized EEG channel locations.

Once we identified the NI potential, we looked at an epoch in the −300 to −50 ms window before the N1 because several previous studies, including those from our laboratory, have reported significant fronto-central negativity (N1) that peaks approximately 100 to 250 ms after the onset of external perturbation [8], [9], [16]–[19], [58]. We took the median of absolute values of the different parameters in the chosen window. First, we looked at basic descriptive statistical comparisons between grand means across all subjects and epochs across the two data sets (*Control* and *Instability*). Results showed significant differences with large effect sizes for three of the four parameters. This may not appear surprising, but this study provides some unique threshold values, beyond which, as per the electrophysiological evidence (N1 potential) that we present, there is a very high chance of triggering a postural instability in our task. For example, based on our results, we can say that if a 250 ms long moving median window detects TTB < 1.5 s (mean TTB of *Instability* data set was 0.49 s, and 3*SD was 3*0.32 s), then, as per the empirical rule, that will effectively cover nearly 100% of all epochs preceding the low-amplitude N1 potentials.

Then we used an advanced machine learning technique to investigate the contributions of different parameters collected before N1s, in predicting the occurrence of N1s. We used a well-established and robust cross-validation technique called leave-one-subject-out. When using all four parameters combined, our results show that TTB was the most important parameter that the model used in predicting the occurrence of low-amplitude N1 potential (Fig. 4). These findings provide stronger evidence that TTB could be the control variable the CNS is tracking for posture control and implement corrective responses if a threshold is breached. Slobounov *et al.* were first to propose that TTB could be the neural detector of postural instability [13], [33], as it combines both spatial and temporal dynamics of postural sway. Various studies over the years, including those from our laboratory, have shown the effectiveness of TTB as a valid measure of postural stability in various populations or scenarios, including older adults [51], [52], people with multiple sclerosis [59], [60], ankle instability [61], Parkinson’s disease [62], virtual temporary lesion [15], as well as in astronauts [39] and single vs. dual-task differences [16], [18]. Postural stability measured by TTB has also been linked to greater fall risk [52], [60], [63]. Similarly, the margin of stability has been shown [64], [65] to be associated with instability during dynamic balance. Gill *et al.* [64] used position-based COM, relative to the base of support (BOS), and tried to predict failed steps during beam walking by classifying COM as inside BOS or outside. They concluded that this classification might be over-simplistic and, in cases where COM was outside BOS, and the subject did not fall. Walking faster helped participants, thus confirming the need to incorporate both position and velocity information, as done by TTB approach used in this study and many others.

In addition, we used each of four parameters independently in the model for the prediction of low-amplitude cortical response following a naturally occurring instability. Our results also showed that when parameters were used independently, COPv was not statistically different than TTB in predicting the occurrence of low-amplitude N1 potential. It should be noted that COPv is one of the components of TTB calculation. Thus, our study provides further evidence that COPv information might be useful for human posture control, as proposed earlier [36], [37]. It is known that the frequency of firing of neurons in the muscle spindles and cutaneous receptors at the sole of the feet encode velocity information [66], [67]. Our results show that even though the somatosensory information was distorted by changing the gain of the sway-referenced support surface, the COPv information was still more relevant than the other two biomechanical parameters (SH and HA), in our task.

When considering model performance using individual parameters, SH was the third most important parameter, and HA was the last (Table I). This is not surprising. This could be because some overlap of the sensory receptors provides information on COPv and SH. The mechanoreceptors at the sole of the feet can be classified into four groups based on the size of the receptive field (type I [small defined boundaries] vs. type II [large undefined boundaries]) and afferent/receptor firing properties (fast adapting [FA] vs. slow adapting [SA]) [68]. FAII receptors, in particular, are known to encode high-frequency vibration information that can signal slips and dynamic contact between the skin and environment, whereas FAI receptors are known to encode tangential (shear) forces [69]. There is some evidence that neighboring neurons may have overlapping receptive fields close to one another [70]. Additionally, Ting *et al.* [34] showed that the shear force change could not solely be the trigger signal for balance correction. They found that the vector direction of shear force biomechanically was opposite for rotation and translation perturbations that generated the same balance response. They suggested that the change in shear to vertical load ratio could be an unambiguous signal that can potentially capture the dynamics (both regarding the rate of change and direction of change) of the disturbance. Similarly, in our study, it is possible that both vertical and tangential components of ground reaction force (when all parameters were combined) provided the most reliable input regarding postural stability. It is also possible that SH could play a more significant role during rapid postural perturbations, unlike the paradigm used in this study.

In the absence of vision and the presence of distorted somatosensory information from the bottom of feet, prior studies have shown that the posture control system reweights sensory information and relies more on the vestibular system [71]. Vestibular sensors provide orientation information about the head concerning the vertical axis and are essential in posture control [72]. However, the performance of the machine learning model using HA in the AP direction alone was typically the worst of all the four parameters. It suggests that the acceleration in AP direction sensed by the vestibular sensors alone was not sufficient to predict cortical involvement in our task. This could be because there is more sensory noise in vestibular signals to detect small changes (as in naturally occurring postural instability) in postural sway as vestibular end-organs have evolved to work with a larger range of sensory inputs (i.e., head movements) [73], [74]. The relative contribution of vestibular signals to posture control during eyes closed is proposed to be not more than 20% [71]. Further, postural responses to sagittal plane translations are not found to be affected by the vestibular loss [75], [76]. However, one may need to analyze head rotation information to better understand the role of vestibular end-organs in tasks like ours.

Our results using leave-one-subject-out cross-validation are encouraging. They show that we can build reliable machine learning models using single epochs to predict outcomes on new subjects. The overall accuracy of around 75% is similar to that obtained by a prior study from our collaborators [21], albeit using a perturbation task. Sensitivity of about 85% and AUC of around 82% are usually considered very good [77].

The study has several strengths. We used individual structural MRI scans and digitized EEG channel locations to create subject-specific head models to identify a fronto-centrally localized independent component resembling the N1 response preceding the functional balance correction. Our MRI-based EEG approach has provided a higher spatial resolution of EEG signals [78]. Importantly, prediction of the occurrence of low-amplitude N1 potential using biomechanical parameters from single epochs, as done in this study, may significantly advance the field of using EEG for predicting upcoming postural instability with just single trials. We acknowledge that we used an independent component created during the post-processing of the entire data. However, it implies that our electrophysiological results are more robust than other studies [14], [19], showing N1 response in electrodes from the fronto-central region for challenging balance tasks.

We do acknowledge that our study has a few limitations. The aim of this paper was not to find an optimal machine learning model or window size; therefore, it is possible that the machine learning performance could be further improved with other more complex machine learning techniques or testing different window sizes. However, it is also possible that some of the computationally heavy machine learning techniques are not suitable for real-time implementation. It is possible that other metrics, for example, variability in the parameters we used, can further improve performance. However, we wanted to keep the parameters simple, as a median filter can be easily implementable on a chip for real-time processing. It is also possible that adding sensors providing information of segmental orientation (e.g., ankle, hip, and knee) could have provided additional information for prediction. These would most likely be available in lower-limb exoskeleton systems and need to be tested in the future. And lastly, we only tried this in a relatively quiet stance. This approach needs to be expanded and tested in more dynamic conditions like walking.

## V. Conclusion

We present a machine learning approach that compared the ability of biomechanical parameters obtained from single epochs to predict the occurrence of low-amplitude N1 potentials during naturally occurring postural instabilities. TTB was found to be the most important biomechanical parameter to predict the occurrence of these low-amplitude N1 potentials when compared with COPv, SH, and HA. These results provide direct evidence suggesting that the spatial and temporal information of the body’s center of pressure is a robust parameter that triggers a cortical sign of postural stability.

## Supplementary Material

supp1-3154707

## Figures and Tables

**TABLE I:** Machine Learning Performance Metrics

Parameter	Accuracy	Sensitivity	AUC

HA	58.0 ±0.8	36.2 ±3.1	60.8 ± 1.0
SH	62.4 ± 0.9	76.2 ±1.6	67.3 ± 1.0
COPv	70.3 ± 0.9	81.2 ± 1.1	76.5 ± 1.0
TTB	70.9 ± 0.5	79.2 ±1.0	77.1 ±0.8
All 4	74.4 ± 0.8	84.2 ±1.1	81.2 ±0.8

Mean ± SE, Chance Was 50%

**TABLE II:** P-Values for Accuracy

Parameter	HA	SH	COPv	TTB

SH	<0.0001*			
COPv	<0.0001*	<0.0001*		
TTB	<0.0001*	<0.0001*	5.3E-01	
All 4	<0.0001*	<0.0001*	<0.0001*	<0.0001*

* Indicates P < 5E-3

**TABLE III:** P-Values for Sensitivity

Parameter	HA	SH	COPv	TTB

SH	<0.0001*			
COPv	<0.0001*	1.1E-02		
TTB	<0.0001*	1.2E-01	1.8E-01	
All 4	<0.0001*	<0.0001*	5.7E-02	<0.0001*

* Indicates P < 5E-3

**TABLE IV:** P-Values for AUC

Parameter	HA	SH	COPv	TTB

SH	<0.0001*			
COPv	<0.0001*	<0.0001*		
TTB	<0.0001*	<0.0001*	6.3E-01	
All 4	<0.0001*	<0.0001*	<0.0001*	<0.0001*

* Indicates P < 5E-3

## References

[1] Rothwell JC, Control of Human Voluntary Movement, 2nd ed. London, U.K.: Chapman & Hall, 1994.

[2] Horak FB and Macpherson JM, “Postural orientation and equilibrium,” in Handbook of Physiology (Exercise: Regulation and Integration of Multiple Systems), Rowell LB and Sheperd JT, Eds. New York, NY, USA: Oxford Univ. Press, 1996, sec. 12, pp. 255–292.

[3] Koozekanani SH, Stockwell CW, McGhee RB, and Firoozmand F, “On the role of dynamic models in quantitative posturography,” IEEE Trans. Biomed. Eng, vol. BE-27, no. 10, pp. 605–609, Oct. 1980. 10.1109/TBME.1980.326583 7439918

[4] Forth KE, Metter EJ, and Paloski WH, “Age associated differences in postural equilibrium control: A comparison between EQscore and minimum time to contact (TTCmin),” Gait Posture, vol. 25, no. 1, pp. 56–62, Jan. 2007. 16464595 10.1016/j.gaitpost.2005.12.008

[5] Dakin CJ and Bolton DAE, “Forecast or fall: Prediction’s importance to postural control,” Frontiers Neurol., vol. 9, p. 924, Oct. 2018. 10.3389/fneur.2018.00924 PMC6218399 30425680

[6] Fujiwara K, Kiyota N, and Maeda K, “Contingent negative variation and activation of postural preparation before postural perturbation by backward floor translation at different initial standing positions,” Neurosci. Lett, vol. 490, pp. 135–139, Feb. 2011. 21185354 10.1016/j.neulet.2010.12.043

[7] Mochizuki G, Boe S, Marlin A, and McIlroy WE, “Perturbationevoked cortical activity reflects both the context and consequence of postural instability,” Neuroscience, vol. 170, no. 2, pp. 599–609, Oct. 2010. 20633605 10.1016/j.neuroscience.2010.07.008

[8] Varghese JP, Marlin A, Beyer KB, Staines WR, Mochizuki G, and McIlroy WE, “Frequency characteristics of cortical activity associated with perturbations to upright stability,” Neurosci. Lett, vol. 578, pp. 8–33, Aug. 2014. 10.1016/j.neulet.2014.06.017 24970752

[9] Marlin A, Mochizuki G, Staines WR, and McIlroy WE, “Localizing evoked cortical activity associated with balance reactions: Does the anterior cingulate play a role?” J. Neurophysiol, vol. 111, pp. 2634–2643, Jun. 2014. 24647435 10.1152/jn.00511.2013

[10] Mierau A, Hülsdünker T, and Strüder HK, “Changes in cortical activity associated with adaptive behavior during repeated balance perturbation of unpredictable timing,” Frontiers Behav. Neurosci, vol. 9, p. 272, Oct. 2015. 10.3389/fnbeh.2015.00272 PMC4604244 26528154

[11] Hülsdünker T, Mierau A, Neeb C, Kleinöder H, and Strüder HK, “Cortical processes associated with continuous balance control as revealed by EEG spectral power,” Neurosci. Lett, vol. 592, pp. 1–5, Apr. 2015. 25724275 10.1016/j.neulet.2015.02.049

[12] Hülsdünker T, Mierau A, and Strüder HK, “Higher balance task demands are associated with an increase in individual alpha peak frequency,” Frontiers Hum. Neurosci, vol. 9, p. 695, Jan. 2016. 10.3389/fnhum.2015.00695 PMC4702132 26779005

[13] Slobounov S, Cao C, Jaiswal N, and Newell KM, “Neural basis of postural instability identified by VTC and EEG,” Exp. Brain Res, vol. 199, no. 1, pp. 1–16, Oct. 2009. 19655130 10.1007/s00221-009-1956-5 PMC2942764

[14] Varghese JP, Beyer KB, Williams L, Miyasike-daSilva V, and McIlroy WE, “Standing still: Is there a role for the cortex?” Neurosci. Lett, vol. 590, pp. 18–23, Mar. 2015. 25623039 10.1016/j.neulet.2015.01.055

[15] Goel R, Nakagome S, Rao N, Paloski WH, Contreras-Vidal JL, and Parikh PJ, “Fronto-parietal brain areas contribute to the online control of posture during a continuous balance task,” Neuroscience, vol. 413, pp. 135–153, Aug. 2019. 31200107 10.1016/j.neuroscience.2019.05.063

[16] Ozdemir RA, Contreras-Vidal JL, and Paloski WH, “Cortical control of upright stance in elderly,” Mech. Ageing Develop, vol. 169, pp. 19–31, Jan. 2018. 10.1016/j.mad.2017.12.004 29277586

[17] Goel R, Ozdemir RA, Nakagome S, Contreras-Vidal JL, Paloski WH, and Parikh PJ, “Effects of speed and direction of perturbation on electroencephalographic and balance responses,” Exp. Brain Res, vol. 236, no. 7, pp. 2073–2083, Jul. 2018. 29752486 10.1007/s00221-018-5284-5

[18] Ozdemir RA, Contreras-Vidal JL, Lee B-C, and Paloski WH, “Cortical activity modulations underlying age-related performance differences during posture–cognition dual tasking,” Exp. Brain Res, vol. 234, no. 11, pp. 3321–3334, Jul. 2016. 27443853 10.1007/s00221-016-4730-5

[19] Ditz JC, Schwarz A, and Müller-Putz GR, “Perturbation-evoked potentials can be classified from single-trial EEG,” J. Neural Eng, vol. 17, no. 3, Jun. 2020, Art. no. 036008. 10.1088/1741-2552/ab89fb 32299075

[20] Gebel A, Lehmann T, and Granacher U, “Balance task difficulty affects postural sway and cortical activity in healthy adolescents,” Exp. Brain Res, vol. 238, no. 5, pp. 1323–1333, May 2020. 32328673 10.1007/s00221-020-05810-1 PMC7237405

[21] Ravindran AS , “Interpretable deep learning models for single trial prediction of balance loss,” in Proc. IEEE Int. Conf. Syst., Man, Cybern. (SMC), Oct. 2020, pp. 268–273.

[22] Solis-Escalante T, De Kam D, and Weerdesteyn V, “Classification of rhythmic cortical activity elicited by whole-body balance perturbations suggests the cortical representation of direction-specific changes in postural stability,” IEEE Trans. Neural Syst. Rehabil. Eng, vol. 28, no. 11, pp. 2566–2574, Nov. 2020. 33021931 10.1109/TNSRE.2020.3028966

[23] Varghese JP, Staines WR, and McIlroy WE, “Activity in functional cortical networks temporally associated with postural instability,” Neuroscience, vol. 401, pp. 43–58, Mar. 2019. 30668974 10.1016/j.neuroscience.2019.01.008

[24] Payne AM, Hajcak G, and Ting LH, “Dissociation of muscle and cortical response scaling to balance perturbation acceleration,” J. Neurophysiol, vol. 121, no. 3, pp. 867–880, Mar. 2019. 30517039 10.1152/jn.00237.2018 PMC6520627

[25] Quinzi F. , “Contribution of cognitive functions to postural control in anticipating self-paced and externally-triggered lower-limb perturbations,” Behav. Brain Res, vol. 366, pp. 56–66, Jul. 2019. 30898679 10.1016/j.bbr.2019.03.033

[26] Edwards AE, Guven O, Furman MD, Arshad Q, and Bronstein AM, “Electroencephalographic correlates of continuous postural tasks of increasing difficulty,” Neuroscience, vol. 395, pp. 35–48, Dec. 2018. 30391529 10.1016/j.neuroscience.2018.10.040

[27] Takakura H, Nishijo H, Ishikawa A, and Shojaku H, “Cerebral hemodynamic responses during dynamic posturography: Analysis with a multichannel near-infrared spectroscopy system,” Frontiers Human Neurosci., vol. 9, p. 620, Nov. 2015. 10.3389/fnhum.2015.00620 PMC4647449 26635574

[28] Slobounov S, Hallett M, Stanhope S, and Shibasaki H, “Role of cerebral cortex in human postural control: An EEG study,” Clin. Neurophysiol, vol. 116, pp. 315–323, Feb. 2005. 15661110 10.1016/j.clinph.2004.09.007

[29] Ahmed AA and Ashton-Miller JA, “Is a ‘loss of balance’ a control error signal anomaly? Evidence for three-sigma failure detection in young adults,” Gait Posture, vol. 19, pp. 252–262, 2004. 15125914 10.1016/S0966-6362(03)00066-3

[30] Jacobs JV and Horak FB, “Cortical control of postural responses,” J. Neural Transmiss, vol. 114, no. 10, pp. 1339–1348, Oct. 2007. 10.1007/s00702-007-0657-0 PMC4382099 17393068

[31] Bolton DA, “The role of the cerebral cortex in postural responses to externally induced perturbations,” Neurosci. Biobehav. Rev, vol. 57, pp. 55–142, Oct. 2015. 10.1016/j.neubiorev.2015.08.014 26321589

[32] Sipp AR, Gwin JT, Makeig S, and Ferris DP, “Loss of balance during balance beam walking elicits a multifocal theta band electrocortical response,” J. Neurophysiol, vol. 110, pp. 2050–2060, Nov. 2013. 23926037 10.1152/jn.00744.2012 PMC3841925

[33] Slobounov SM, Slobounov ES, and Newell KM, “Virtual timeto-collision and human postural control,” J. Motor Behav, vol. 29, pp. 263–281, Sep. 1997. 10.1080/00222899709600841 12453785

[34] Ting LH and Macpherson JM, “Ratio of shear to load ground-reaction force may underlie the directional tuning of the automatic postural response to rotation and translation,” J. Neurophysiol, vol. 92, pp. 808–823, Aug. 2004. 15084643 10.1152/jn.00773.2003

[35] Yu E. , “Evaluation of postural control in quiet standing using center of mass acceleration: Comparison among the young, the elderly, and people with stroke,” Arch. Phys. Med. Rehabil, vol. 89, pp. 1133–1139, Jun. 2008. 18503811 10.1016/j.apmr.2007.10.047

[36] Masani K, Popovic MR, Nakazawa K, Kouzaki M, and Nozaki D, “Importance of body sway velocity information in controlling ankle extensor activities during quiet stance,” J. Neurophysiol, vol. 90, pp. 3774–3782, Dec. 2003. 12944529 10.1152/jn.00730.2002

[37] Jeka J, Kiemel T, Creath R, Horak F, and Peterka R, “Controlling human upright posture: Velocity information is more accurate than position or acceleration,” J. Neurophysiol, vol. 92, pp. 2368–2379, Oct. 2004. 15140910 10.1152/jn.00983.2003

[38] Wood SJ, Paloski WH, and Clark JB, “Assessing sensorimotor function following ISS with computerized dynamic posturography,” Aerosp. Med. Hum. Perform, vol. 86, no. 12, pp. 45–53, Dec. 2015. 10.3357/AMHP.EC07.2015 26630195

[39] Ozdemir RA, Goel R, Reschke MF, Wood SJ, and Paloski WH, “Critical role of somatosensation in postural control following spaceflight: Vestibularly deficient astronauts are not able to maintain upright stance during compromised somatosensation,” Frontiers Physiol., vol. 9, p. 1680, Nov. 2018. 10.3389/fphys.2018.01680 PMC6277541 30538640

[40] Cohen HS and Kimball KT, “Usefulness of some current balance tests for identifying individuals with disequilibrium due to vestibular impairments,” J. Vestibular Res, vol. 18, pp. 295–303, Jan. 2008. PMC2819299 19542603

[41] Luu TP, He Y, Brown S, Nakagome S, and Contreras-Vidal JL, “Gait adaptation to visual kinematic perturbations using a real-time closed-loop brain–computer interface to a virtual reality avatar,” J. Neural Eng, vol. 13, no. 3, Jun. 2016, Art. no. 036006. 10.1088/1741-2560/13/3/036006 PMC5726869 27064824

[42] Clinical Interpretation Guide—Computerized Dynamic Posturography, Neurocom, Pleasanton, CA, USA, 2009.

[43] Ozdemir RA, Pourmoghaddam A, and Paloski WH, “Sensorimotor posture control in the blind: Superior ankle proprioceptive acuity does not compensate for vision loss,” Gait Posture, vol. 38, pp. 603–608, Sep. 2013. 23477840 10.1016/j.gaitpost.2013.02.003

[44] Bigdely-Shamlo N, Mullen T, Kothe C, Su K-M, and Robbins KA, “The PREP pipeline: Standardized preprocessing for large-scale EEG analysis,” Frontiers Neuroinform., vol. 9, p 16, Jun. 2015. 10.3389/fninf.2015.00016 PMC4471356 26150785

[45] Mullen T. , “Real-time modeling and 3D visualization of source dynamics and connectivity using wearable EEG,” in Proc. 35th Annu. Int. Conf. IEEE Eng. Med. Biol. Soc. (EMBC), Jul. 2013, pp. 2184–2187. 10.1109/EMBC.2013.6609968 PMC4119601 24110155

[46] Palmer JA, Kreutz-Delgado K, and Makeig S, “AMICA: An adaptive mixture of independent component analyzers with shared components,” Swartz Center Comput. Neurosci, San Diego, CA, USA, Tech. Rep., 2011.

[47] Todd NPM, Paillard AC, Kluk K, Whittle E, and Colebatch JG, “Source analysis of short and long latency vestibular-evoked potentials (VsEPs) produced by left vs. right ear air-conducted 500 Hz tone pips,” Hearing Res., vol. 312, pp. 91–102, Jun. 2014. 10.1016/j.heares.2014.03.006 PMC4017095 24699384

[48] Chen T. and Guestrin C, “XGBoost: A scalable tree boosting system,” in Proc. 22nd ACM SIGKDD Int. Conf. Knowl. Discovery Data Mining, San Francisco, CA, USA, Aug. 2016, pp. 785–794.

[49] Kamogashira T, Fujimoto C, Kinoshita M, Kikkawa Y, Yamasoba T, and Iwasaki S, “Prediction of vestibular dysfunction by applying machine learning algorithms to postural instability,” Frontiers Neurol., vol. 11, p. 7, Feb. 2020. 10.3389/fneur.2020.00007 PMC7013037 32116997

[50] Lundberg SM , “From local explanations to global understanding with explainable AI for trees,” Nature Mach. Intell, vol. 2, no. 1, pp. 56–67, 2020. 32607472 10.1038/s42256-019-0138-9 PMC7326367

[51] Slobounov SM, Moss SA, Slobounova ES, and Newell KM, “Aging and time to instability in posture,” J. Gerontol. A, Biol. Sci. Med. Sci, vol. 53A, no. 1, pp. B71–B80, Jan. 1998. 10.1093/gerona/53a.1.b71 9467425

[52] Hsieh KL, Moon Y, Ramkrishnan V, Ratnam R, and Sosnoff JJ, “Validating virtual time to contact with home-based technology in young and older adults,” J. Appl. Biomechanics, vol. 35, no. 1, pp. 61–67, Feb. 2019. 10.1123/jab.2018-0088 30207197

[53] Ackermann H, Diener HC, and Dichgans J, “Mechanically evoked cerebral potentials and long-latency muscle responses in the evaluation of afferent and efferent long-loop pathways in humans,” Neurosci. Lett, vol. 66, pp. 233–238, May 1986. 3725188 10.1016/0304-3940(86)90024-8

[54] Payne AM and Ting LH, “Balance perturbation-evoked cortical N1 responses are larger when stepping and not influenced by motor planning,” J. Neurophysiol, vol. 124, no. 6, pp. 1875–1884, Dec. 2020. 33052770 10.1152/jn.00341.2020 PMC7814905

[55] Duckrow RB, Abu-Hasaballah K, Whipple R, and Wolfson L, “Stance perturbation-evoked potentials in old people with poor gait and balance,” Clin. Neurophysiol, vol. 110, pp. 32–2026, Dec. 1999. 10.1016/s1388-2457(99)00195-9 10616107

[56] Cioncoloni D. , “Role of brain hemispheric dominance in anticipatory postural control strategies,” Exp. Brain Res, vol. 234, pp. 1997–2005, Jul. 2016. 26952051 10.1007/s00221-016-4603-y

[57] Taubert M. , “Dynamic properties of human brain structure: Learning-related changes in cortical areas and associated fiber connections,” J. Neurosci, vol. 30, pp. 11670–11677, Sep. 2010. 20810887 10.1523/JNEUROSCI.2567-10.2010 PMC6633410

[58] Maki BE and McIlroy WE, “Cognitive demands and cortical control of human balance-recovery reactions,” J. Neural Transmiss, vol. 114, pp. 1279–1296, Oct. 2007. 10.1007/s00702-007-0764-y 17557125

[59] Whittier TT, Richmond SB, Monaghan AS, and Fling BW, “Virtual time-to-contact identifies balance deficits better than traditional metrics in people with multiple sclerosis,” Exp. Brain Res, vol. 238, no. 1, pp. 93–99, Jan. 2020. 31792556 10.1007/s00221-019-05698-6

[60] Cattaneo D, Ferrarin M, Jonsdottir J, Montesano A, and Bove M, “The virtual time to contact in the evaluation of balance disorders and prediction of falls in people with multiple sclerosis,” Disab. Rehabil, vol. 34, pp. 470–477, Mar. 2012. 10.3109/09638288.2011.608144 21985076

[61] Hertel J, Olmsted-Kramer LC, and Challis JH, “Time-to-boundary measures of postural control during single leg quiet standing,” J. Appl. Biomech, vol. 22, no. 1, pp. 67–73, Feb. 2006. 16760569 10.1123/jab.22.1.67

[62] van Wegen EEH, van Emmerik REA, and Riccio GE, “Postural orientation: Age-related changes in variability and time-to-boundary,” Hum. Movement Sci, vol. 21, no. 1, pp. 61–84, Apr. 2002. 10.1016/s0167-9457(02)00077-5 11983434

[63] Kilby MC, Slobounov SM, and Newell KM, “Aging and the recovery of postural stability from taking a step,” Gait Posture, vol. 40, no. 4, pp. 701–706, Sep. 2014. 25161010 10.1016/j.gaitpost.2014.08.002

[64] Gill L, Huntley AH, and Mansfield A, “Does the margin of stability measure predict medio-lateral stability of gait with a constrained-width base of support?” J. Biomech, vol. 95, Oct. 2019, Art. no. 109317. 10.1016/j.jbiomech.2019.109317 31466717

[65] Hak L, Hettinga FJ, Duffy KR, Jackson J, Sandercock GRH, and Taylor MJD, “The concept of margins of stability can be used to better understand a change in obstacle crossing strategy with an increase in age,” J. Biomech, vol. 84, pp. 147–152, Feb. 2019. 30642664 10.1016/j.jbiomech.2018.12.037

[66] Welch TD and Ting LH, “A feedback model reproduces muscle activity during human postural responses to support-surface translations,” J. Neurophysiol, vol. 99, pp. 1032–1038, Feb. 2008. 18094102 10.1152/jn.01110.2007

[67] Peters RM, Dalton BH, Blouin J-S, and Inglis JT, “Precise coding of ankle angle and velocity by human calf muscle spindles,” Neuroscience, vol. 349, pp. 98–105, May 2017. 28263787 10.1016/j.neuroscience.2017.02.034

[68] Lowrey CR, Perry SD, Strzalkowski ND, Williams DR, Wood SJ, and Bent LR, “Selective skin sensitivity changes and sensory reweighting following short-duration space flight,” J. Appl. Physiol, vol. 116, pp. 683–692, Mar. 2014. 24458748 10.1152/japplphysiol.01200.2013

[69] Macefield VG, “Physiological characteristics of low-threshold mechanoreceptors in joints, muscle and skin in human subjects,” Clin. Exp. Pharmacol. Physiol, vol. 32, pp. 135–144, Jan./Feb. 2005. 15730450 10.1111/j.1440-1681.2005.04143.x

[70] Bentley NM and Salinas E, “Neural coding of spatial representations,” in Encyclopedia of Neuroscience, vol. 6, Squire LR, Ed. Amsterdam, The Netherlands: Elsevier, 2009, pp. 117–122.

[71] Peterka RJ, “Sensorimotor integration in human postural control,” J. Neurophysiol, vol. 88, no. 3, pp. 1097–1118, Sep. 2002. 12205132 10.1152/jn.2002.88.3.1097

[72] Horak FB, “Postural orientation and equilibrium: What do we need to know about neural control of balance to prevent falls?” Age Ageing, vol. 35, no. 2, pp. 7–11, Sep. 2006. 10.1093/ageing/afl077 16926210

[73] Faisal AA, Selen LPJ, and Wolpert DM, “Noise in the nervous system,” Nature Rev. Neurosci, vol. 9, no. 4, pp. 292–303, Apr. 2008. 18319728 10.1038/nrn2258 PMC2631351

[74] van der Kooij H. and Peterka RJ, “Non-linear stimulus-response behavior of the human stance control system is predicted by optimization of a system with sensory and motor noise,” J. Comput. Neurosci, vol. 30, pp. 759–778, Jun. 2011. 21161357 10.1007/s10827-010-0291-y PMC3108015

[75] Horak FB, Nashner LM, and Diener HC, “Postural strategies associated with somatosensory and vestibular loss,” Exp. Brain Res, vol. 82, no. 1, pp. 77–167, Aug. 1990. 2257901 10.1007/BF00230848

[76] Runge CF, Shupert CL, Horak FB, and Zajac FE, “Role of vestibular information in initiation of rapid postural responses,” Exp. Brain Res, vol. 122, pp. 403–412, Oct. 1998. 9827859 10.1007/s002210050528

[77] Hosmer DW, Lemeshow S, and Strudivant RX, “Assessing the fit of the model,” in Applied Logistic Regression, 3rd ed. New York, NY, USA: Wiley, 2013, pp. 173–181.

[78] Acar Z. and Makeig S, “Effects of forward model errors on EEG source localization,” Brain Topogr., vol. 26, no. 3, pp. 378–396, Jul. 2013. 23355112 10.1007/s10548-012-0274-6 PMC3683142

