# Supplementary material for: Assessment of Biomechanical Predictors of Occurrence of Low-Amplitude N1 Potentials Evoked by Naturally Occurring Postural Instabilities
Source: IEEE Trans Neural Syst Rehabil Eng. Author manuscript; Available in PMC 2024 Apr 26. (PMC11047164; doi:10.1109/TNSRE.2022.3154707)
Supplement: supp1-3154707 [file NIHMS1850164-supplement-supp1-3154707.docx]

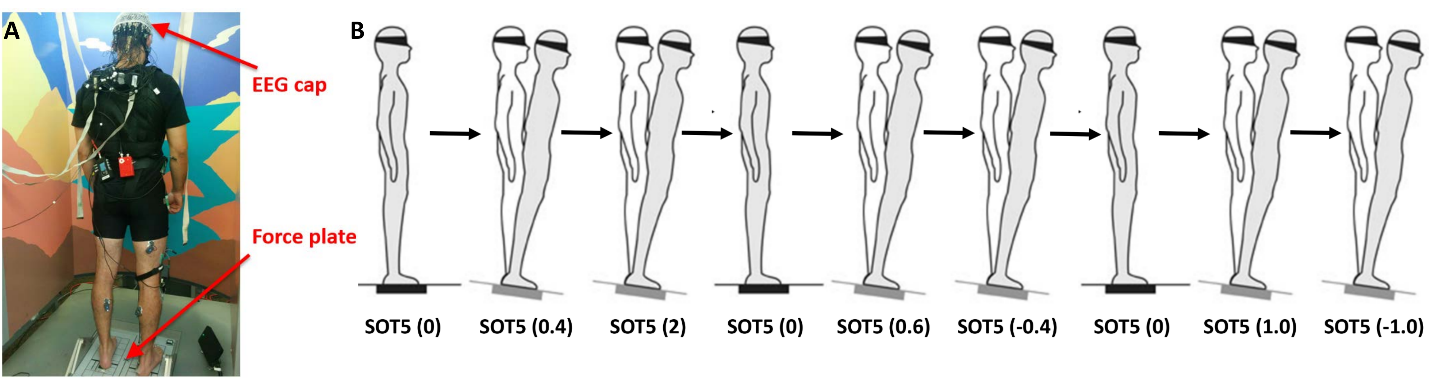


Fig. S1. Experimental setup and the balance task. A. Experimental setup for the posture task. B. Continuous balance task. Schematic description of experimental conditions during the continuous balance task. Three conditions were performed for the support surface gain of 0, whereas one condition each was performed for six other support surface gain values (−1.0, −0.4, 0.4, 0.6, 1.0, 2.0). This is same as Fig. 1 in our previous publication. Reprinted by permission from Elsevier: [Neuroscience] [Fronto-Parietal Brain Areas Contribute to the Online Control of Posture during a Continuous Balance Task, Goel R, Nakagome S, Rao N, Paloski WH, Contreras-Vidal JL, Parikh PJ] [COPYRIGHT] (2019)


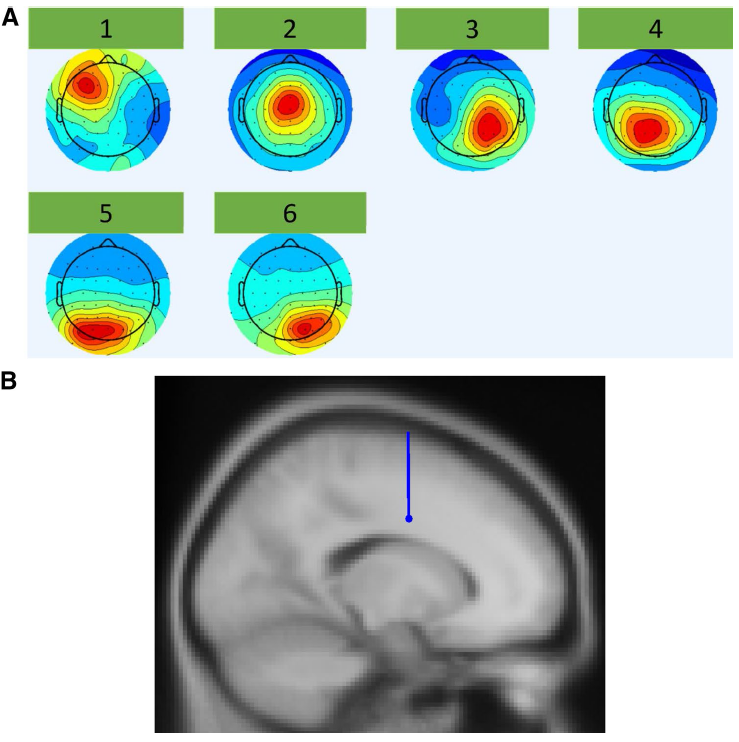


Fig. S2. A Two-dimensional topoplots of independent components (ICs) for a representative subject. These topoplots suggested that IC2 (blue) shows the fronto-central activity. B Sagittal view of IC2 (blue) which was selected for this subject as this was originating in the fronto-central region. This is same as Fig. 2 in our previous publication. Reprinted by permission from Springer Nature: [Experimental Brain Research] [Effects of speed and direction of perturbation on electroencephalographic and balance responses, Goel R, Ozdemir,RA, Nakagome S, Contreras-Vidal JL, Paloski WH, Parikh PJ] [COPYRIGHT] (2018)


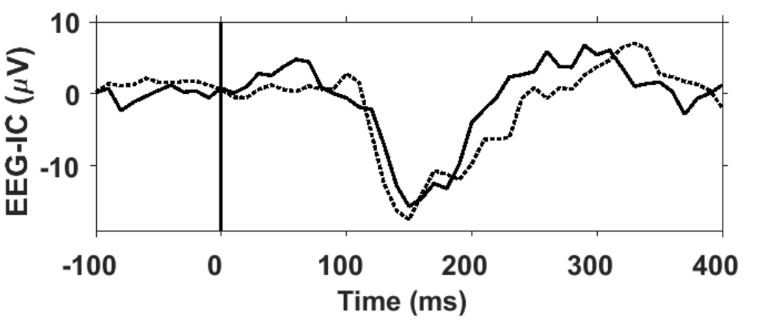


Fig. S3. Time series of the IC from the fronto-central region for the first two perturbation trials. The solid vertical line represents the onset of external perturbation. This is same as part of Fig. 4A in our previous publication. Reprinted by permission from Springer Nature: [Experimental Brain Research] [Effects of speed and direction of perturbation on electroencephalographic and balance responses, Goel R, Ozdemir,RA, Nakagome S, Contreras-Vidal JL, Paloski WH, Parikh PJ] [COPYRIGHT] (2018)
